# One Adaptive Synchronization Approach for Fractional-Order Chaotic System with Fractional-Order 1 < *q* < 2

**DOI:** 10.1155/2014/490364

**Published:** 2014-08-27

**Authors:** Ping Zhou, Rongji Bai

**Affiliations:** ^1^Center of System Theory and Its Applications, Chongqing University of Posts and Telecommunications, Chongqing 400065, China; ^2^Key Laboratory of Network Control and Intelligent Instrument of Ministry of Education, Chongqing University of Posts and Telecommunications, Chongqing 400065, China

## Abstract

Based on a new stability result of equilibrium point in nonlinear fractional-order systems for fractional-order lying in 1 < *q* < 2, one adaptive synchronization approach is established. The adaptive synchronization for the fractional-order Lorenz chaotic system with fractional-order 1 < *q* < 2 is considered. Numerical simulations show the validity and feasibility of the proposed scheme.

## 1. Introduction

Fractional-order differential equations can be more accurately described in the real-world physical systems [1–3]. Many fractional-order systems create chaotic attractor. Many fractional-order chaotic attractors have been reported in recent years, for example, the fractional-order Lorenz chaotic attractor [1, 4, 5], the fractional-order Chen chaotic attractor [5], the fractional-order Lu chaotic attractor [2, 4], the fractional-order Chua chaotic attractor [5], the fractional-order Duffing chaotic attractor [6], the fractional-order Rössler chaotic attractor [7, 8], and so on. On the other hand, synchronization of chaotic systems has been given more attention [2, 9–13]. This is due to its applications in the field of engineering and science. Over the last two decades, many scholars have proposed various synchronization schemes. It is well known that many chaotic systems in practical situations are usually with fully or partially unknown parameters. In order to estimate the unknown parameters, a synchronization scheme named adaptive synchronization has been proposed. Now, the adaptive synchronization [13–16] has attracted more and more attention. This is due to its effectiveness in many practical chaos applications.

However, many adaptive synchronization approaches on fractional-order chaotic systems reported previously [2, 9–13] were considered the fractional-order lying in 0 < *q* < 1. To the best of our knowledge, there are a few results about adaptive synchronization on fractional-order chaotic systems with fractional-order  1 < *q* < 2. In fact, there are many fractional-order systems with fractional-order 1 < *q* < 2 in real-world physical systems, for example, the fractional diffusion-wave equation [17], the fractional telegraph equation [18], the time fractional heat conduction equation [19], and so forth. So, an interesting question is how to realize the adaptive synchronization for fractional-order lying in 1 < *q* < 2? This question is of practical importance as well as academic significance. In this paper, a positive answer is given for the above question.

Inspired by the above-mentioned discussion, one adaptive synchronization approach for a class of fractional-order chaotic system with 1 < *q* < 2 is established. This approach is based on a new stability result of equilibrium point in nonlinear fractional-order systems for fractional-order lying in 1 < *q* < 2 [1]. The adaptive synchronization for the fractional-order Lorenz chaotic system with fractional-order 1 < *q* < 2 is considered. Numerical simulations show the validity and feasibility of the proposed scheme.

## 2. Preliminaries and Main Results

In our paper, the *q*th Caputo derivative for function *g*(*t*) is shown as

(1) Dqg(t)=1Γ(l−q)∫0tg(l)(τ)(t−τ)q+1−ldτ, l−1<q<l,

where *D* ^*q*^ denote the Caputo derivative, *l* is the smallest integer larger than *q*, *g* ^(*l*)^(*t*) is the *l* th derivative in the usual sense, and Γ is the gamma function.

Now, consider the following fractional-order chaotic system:

(2) $$\begin{array}{c} D^{q}x=f\left(x\right)=Mx+n\left(x\right), \end{array}$$

where fractional-order 1 < *q* < 2, *x* ∈ *R* ^*n*×1^, and *f*(*x*) ∈ *R* ^*n*×1^. *M* ∈ *R* ^*n*×*n*^ is a constant matrix. *n*(*x*) ∈ *R* ^*n*×1^ is the nonlinear part of system (2).

The system (2) can be rewritten as follows:

(3) $$\begin{array}{c} D^{q}x=L\left(\begin{array}{c} x\\ \sigma_{0} \end{array}\right)+n\left(x,\sigma_{0}\right), \end{array}$$

where *σ*_0_ ∈ *R* is the system parameter. *n*(*x*, *σ*_0_) ∈ *R* ^*n*×1^ is the nonlinear part, and all the terms with system parameter *σ*_0_ are contained in *n*(*x*, *σ*_0_). *L* ∈ *R* ^*n*×(*n*+1)^ is a constant matrix, and matrix element *L*_*i*,*n*+1_ = 0  (*i* = 1,2,…, *n*).

In this paper, we focus on a class of fractional-order chaotic system in which the equation *n*(*y*, *σ*_0_) − *n*(*x*, *σ*_0_) = *n*_*lp*_(*x*)(*y* − *x*) + *n*_*np*_(*y* − *x*, *x*) holds. Here, variable *y* ∈ *R* ^*n*×1^ is real number. Vector *n*_*lp*_(*x*)(*y* − *x*) and vector *n*_*np*_(*y* − *x*, *x*) are the linear part and nonlinear part with respect to (*y* − *x*), respectively. In fact, the nonlinear term *n*(*x*, *σ*_0_) in many fractional-order chaotic systems meet this equation, for example, the fractional-order Lorenz chaotic system, fractional-order Chen chaotic system, fractional-order Lu chaotic system, fractional-order Rössler chaotic system, the fractional-order Chua's chaotic system and its modified chaotic system, the fractional-order Duffing chaotic system, the fractional-order Arneodo chaotic system, the fractional-order Sprott chaotic system, and so forth.

Next, the adaptive synchronization for fractional-order chaotic system (3) is proposed. Select system (3) as drive system; the response systems with parameter update law are shown as follows:

(4) Dqy=L(yσ)+n(y,σ)+u(x,y,σ),Dqσ=Ωe,

where *y* ∈ *R* ^*n*×1^ is state vector, *u*(*x*, *y*, *σ*) ∈ *R* ^*n*×1^ is a controller, *Ω* ∈ *R* ^1×(*n*+1)^ is real constant matrix, and parameter *σ* is unknown in response system (4). The true value of the “unknown” parameter *σ* is selected as *σ*_0_. The parameter update law is *D* ^*q*^ *σ* = *Ωe*. The adaptive synchronization errors are *e* = (*e*_1_,…, *e*_*n*_, *e*_*n*+1_)^**T**^ ∈ *R* ^(*n*+1)×1^, *e*_*i*_ = (*y*_*i*_ − *x*_*i*_) ∈ *R*  (*i* = 1,2,…, *n*), and *e*_*n*+1_ = *e*_*σ*_ = (*σ* − *σ*_0_) ∈ *R*.

**Lemma 1 (see [1]).**

For the nonlinear part *n*(*x*) of systems (2), if

- *n*(*x*)|_*x*=0_ = 0, lim⁡_*x*→0_(||*n*(*x*)||/||*x*||) = 0;
- *Re*[*λ*(*M*)] < 0, −max⁡[*Reλ*(*M*)]>[Γ(*q*)]^1/*q*^.

Then, the zero solution of fractional-order chaotic system (2) is asymptotically stable.

Based on this lemma, the following main results are given.

**Theorem 2 .**

If the controller is selected as

(5) $$\begin{array}{c} u\left(x,y,\sigma \right)=\left[F-n_{lp}\left(x\right)\right]e \end{array}$$

and the following conditions are satisfied:

- ${\left(\begin{array}{c} n_{np}(e,x)\\ 0 \end{array}\right)|}_{e=0}=0$, $\lim_{e\to 0}\left(||\left(\begin{array}{c} n_{np}(e,x)\\ 0 \end{array}\right)||/||e||\right)=0$ for any *x*,
- Re[λ(L+FΩ)]<0, -max⁡[Reλ(L+FΩ)]>[Γ(q)]1/q,

then the adaptive synchronization between fractional-order chaotic system (3) and fractional-order system (4) can be arrived, where *n*(*y*, *σ*) − *n*(*x*, *σ*_0_) = *n*_*lp*_(*x*)*e* + *n*_*np*_(*e*, *x*), *n*_*lp*_(*x*) ∈ *R* ^*n*×(*n*+1)^, and *n*_*np*_(*e*, *x*) ∈ *R* ^*n*×1^. *F* ∈ *R* ^*n*×(*n*+1)^ is a suitable constant matrix.

**Proof**

The error system between systems (4) and (3) can be shown as

(6) Dq(y−x)=L((yσ)−(xσ0))+n(y,σ)−n(x,σ0)+u(x,y,σ).Dqσ=Ωe.

Due to *e* = (*e*_1_,…, *e*_*n*_, *e*_*n*+1_)^**T**^, *e*_*i*_ = *y*_*i*_ − *x*_*i*_  (*i* = 1,2,…, *n*), and *e*_*n*+1_ = *e*_*σ*_ = (*σ* − *σ*_0_), system (6) can be rewritten as

(7) Dq(y−x)=Le+n(y,σ)−n(x,σ0)+u(x,y,σ),Dqσ=Ωe.

Using *n*(*y*, *σ*) − *n*(*x*, *σ*_0_) = *n*_*lp*_(*x*)*e* + *n*_*np*_(*e*, *x*), *D* ^*q*^ *σ*_0_ = 0, and *D* ^*q*^ *σ* = *D* ^*q*^(*σ* − *σ*_0_) = *D* ^*q*^ *e*_*σ*_, error system (7) can be changed as

(8) Dq(y−x)=Le+nlp(x)e+nnp(e,x)+u(x,y,σ),Dqeσ=Ωe.

Since *u*(*x*, *y*, *σ*) = [*F* − *n*_*lp*_(*x*)]*e* and *e* = (*e*_1_,…, *e*_*n*_, *e*_*n*+1_)^**T**^, therefore, (8) can be changed as

(9) $$\begin{array}{c} D^{q}e=\left(\begin{array}{c} L+F\\ \Omega \end{array}\right)e+\left(\begin{array}{c} n_{np}\left(e,x\right)\\ 0 \end{array}\right). \end{array}$$

Due to ${\left(\begin{array}{c} n_{np}(e,x)\\ 0 \end{array}\right)|}_{e=0}=0$, $\lim_{e\to 0}\left(||\left(\begin{array}{c} n_{np}(e,x)\\ 0 \end{array}\right)||/||e||\right)=0$ for any *x*, Re[λ(L+FΩ)]<0, and -max⁡[Reλ(L+FΩ)]>[Γ(q)]1/q. According to the above-mentioned lemma, the zero solution of fractional-order system (9) is asymptotically stable. So, the following result holds:

(10) $$\begin{array}{c} \underset{t\to +\infty }{\lim}||e||=0. \end{array}$$

It implies the following:

(11) lim⁡t→+∞||y−x||=0,  lim⁡t→+∞(σ−σ0)=0.

Therefore, the adaptive synchronization between fractional-order chaotic system (3) and fractional-order system (4) can be arrived. The proof is completed.

## 3. Illustrative Example

In this section, to show the effectiveness of the adaptive synchronization approach in this paper, the adaptive synchronization for the fractional-order Lorenz chaotic system [4] with fractional-order 1 < *q* < 2 is considered. Numerical simulations show the validity and feasibility of the proposed scheme.

The fractional-order Lorenz system is described by

**Figure 1:**
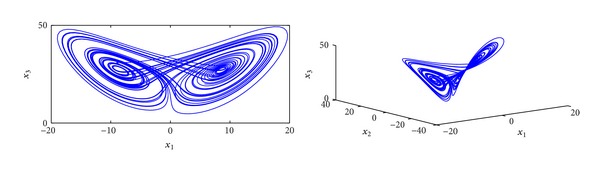
The attractor of fractional-order Lorenz system (12) for *q* = 1.05.

(12) (Dqx1Dqx2Dqx3)=(a0(x2−x1)b0x1−x2−x1x3x1x2−c0x3),

where *a*_0_, *b*_0_, and *c*_0_ are system parameters. Let *a*_0_ = 10, *b*_0_ = 28, *c*_0_ = 8/3, and *q* = 1.05; the system (12) creates chaotic attractor. The chaotic attractor is shown in Figure 1.

**Case 1 (parameter *a* is unknown in response system).**

Now, assume the system parameter  *a*  in in response system is the unknown parameter. The true value of the “unknown” parameter *a* is selected as *a*_0_.

The fractional-order Lorenz system (12) can be rewritten as

(13) (Dqx1Dqx2Dqx3)=(000028−10000−830)(x1x2x3a0)+(a0(x2−x1)−x1x3x1x2),

where *a*_0_ = 10.

So

(14) L=(000028−10000−830).

According to Section 2, the response system with unknown parameter *a* can be given as

(15) (Dqy1Dqy2Dqy3)=(000028−10000−830)(y1y2y3a)+(a(y2−y1)−y1y3y1y2)+(F−nlp(x))(e1e2e3ea)

and the parameter update law is

(16) $$\begin{array}{c} D^{q}a=\Omega {\left(\begin{array}{cccc} e_{1} & e_{2} & e_{3} & e_{a} \end{array}\right)}^{T}. \end{array}$$

It is easy to obtain the following:

(17) (a(y2−y1)−y1y3y1y2)−(a0(x2−x1)−x1x3x1x2)  =(−a0a00x2−x1−x30−x10x2x100)(e1e2e3ea)   +(ea(e2−e1)−e1e3e1e2).

So

(18) $$\begin{array}{c} n_{lp}\left(x\right)=\left(\begin{array}{cccc} -a_{0} & a_{0} & 0 & x_{2}-x_{1}\\ -x_{3} & 0 & -x_{1} & 0\\ x_{2} & x_{1} & 0 & 0 \end{array}\right),\\ n_{np}\left(e,x\right)=\left(\begin{array}{c} e_{a}\left(e_{2}-e_{1}\right)\\ -e_{1}e_{3}\\ e_{1}e_{2} \end{array}\right). \end{array}$$

Now, it is easy to verify the following:

(19) ||(nnp(e,x)0)||||e||  =ea2(e2−e1)2+(e1e3)2+(e1e2)2e12+e22+e32+ea2  ≤ea2(|e2|+|e1|)2+(e1e3)2+(e1e2)2e12+e22+e32+ea2  =ea2(|e2|+|e1|)2e12+e22+e32+ea2+(e1e3)2+(e1e2)2e12+e22+e32+ea2  ≤ea2(|e2|+|e1|)2ea2+(e1e3)2+(e1e2)2e12  =(|e2|+|e1|)2+(e2)2+(e3)2,lim⁡e→0||(nnp(e,x)0)||||e||  ≤lim⁡e→0(|e2|+|e1|)2+(e2)2+(e3)2=0,(nnp(e,x)0)|e=0=0.

Therefore, the first condition in the above-mentioned theorem holds.

Now, choose suitable real constant matrix *Ω* ∈ *R* ^1×*n*^ and *F* ∈ *R* ^*n*×(*n*+1)^ such that

(20) Re[λ(L+FΩ)]<0,−max⁡[Reλ(L+FΩ)]>[Γ(q)]1/q.

So the second condition in the above-mentioned theorem holds. According to the theorem in Section 2, the adaptive synchronization between drive system (13) and response system (15) with parameter update law (16) can be achieved.

**Figure 2:**
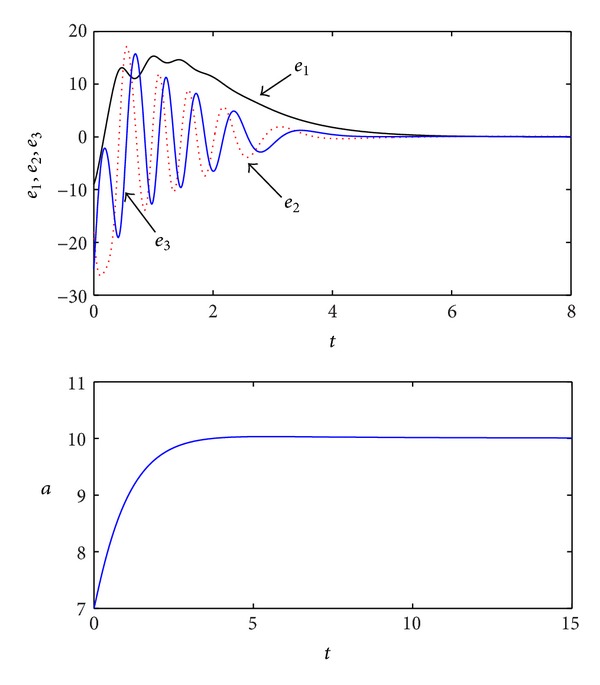
Simulation results of the adaptive synchronization for the fractional-order Lorenz chaotic system.

For example, let $F=\left(\begin{array}{cccc} -1 & 0 & 0 & 0\\ -28 & 0 & 0 & 0\\ 0 & 0 & 0 & 0 \end{array}\right)$ and $\Omega =\left(\begin{array}{cccc} 0 & 0 & 0 & -1 \end{array}\right)$. So $\left(\begin{array}{c} L+F\\ \Omega \end{array}\right)=\left(\begin{array}{cccc} -1 & 0 & 0 & 0\\ 0 & -1 & 0 & 0\\ 0 & 0 & -8/3 & 0\\ 0 & 0 & 0 & -1 \end{array}\right)$. Therefore, *λ*_*i*_ = −1  (*i* = 1,2, 3), *λ*_4_ = −8/3, and -max⁡[Reλ(L+FΩ)]=1>[Γ(q)]1/q=0.9722, respectively. Simulation results are shown in Figure 2. Here, *a*(0) = 7, and all the initial conditions in this paper are (*x*_10_, *x*_20_, *x*_30_) = (10,20,30), and (*y*_10_, *y*_20_, *y*_30_) = (1,2, 5), respectively.

**Case 2 (parameter *b* is unknown in response system).**

Now, assume that the system parameter *b* in response system is the unknown parameter. The true value of the “unknown” parameter *b* is selected as *b*_0_.

The fractional-order Lorenz system (12) can be rewritten as

(21) (Dqx1Dqx2Dqx3)=(−1010000−10000−830)(x1x2x3b0)+(0b0x1−x1x3x1x2),

where *b*_0_ = 28.

So

(22) L=(−1010000−10000−830).

According to Section 2, the response system with unknown parameter *b* can be given as

(23) (Dq2y1Dq2y2Dq2y3)=(−1010000−10000−830)(y1y2y3b)+(0by1−y1y3y1y2)+(F−nlp(x))(e1e2e3eb)

and the parameter update law is

(24) $$\begin{array}{c} D^{q}b=\Omega {\left(\begin{array}{cccc} e_{1} & e_{2} & e_{3} & e_{b} \end{array}\right)}^{T}. \end{array}$$

It is easy to obtain the following:

(25) (0by1−y1y3y1y2)−(0b0x1−x1x3x1x2)  =(0000b0−x30−x10x2x100)(e1e2e3eb)   +(0−e1e3+ebe1e1e2).

So

(26) nlp(x)=(0000b0−x30−x10x2x100),nnp(e,x)=(0−e1e3+ebe1e1e2).

Now, it is easy to verify the following:

(27) ||(nnp(e,x)0)||||e||  =(−e1e3+ebe1)2+(e1e2)2e12+e22+e32+eb2  ≤(|e3|+|eb|)2e12+(e1e2)2e12+e22+e32+eb2  ≤(|e3|+|eb|)2e12+(e1e2)2e12  =(|e3|+|eb|)2+(e2)2,lim⁡e→0||(nnp(e,x)0)||||e||≤lim⁡e→0(|e3|+|eb|)2+(e2)2=0,(nnp(e,x)0)|e=0=0.

Therefore, the first condition in the above-mentioned theorem holds.

Now, choose suitable real constant matrix *Ω* ∈ *R* ^1×*n*^ and *F* ∈ *R* ^*n*×(*n*+1)^ such that

(28) Re[λ(L+FΩ)]<0,−max⁡[Reλ(L+FΩ)]>[Γ(q)]1/q.

So the second condition in the above-mentioned theorem holds. According to the theorem in Section 2, the adaptive synchronization between drive system (21) and response system (23) with parameter update law (24) can be achieved.

**Figure 3:**
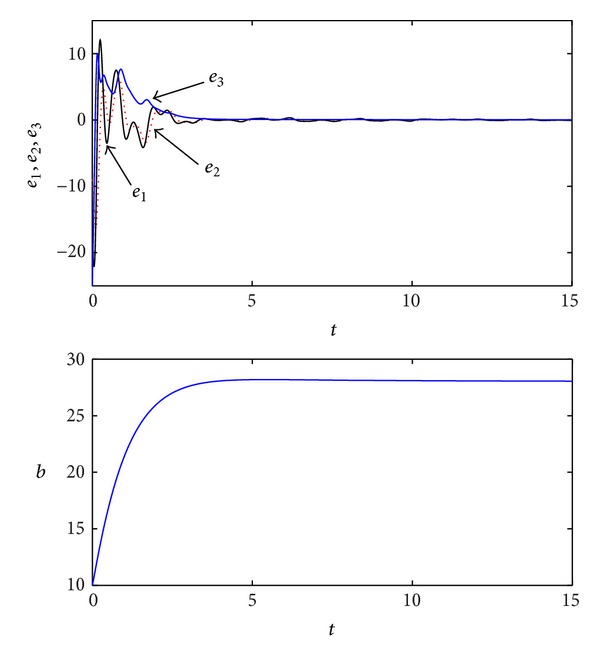
Simulation results of the adaptive synchronization for the fractional-order Lorenz chaotic system.

For example, let $F=\left(\begin{array}{cccc} 0 & 0 & 0 & 0\\ -10 & 0 & 0 & 0\\ 0 & 0 & 0 & 0 \end{array}\right)$ and $\Omega =\left(\begin{array}{cccc} 0 & 0 & 0 & -1 \end{array}\right)$. So $\left(\begin{array}{c} L+F\\ \Omega \end{array}\right)=\left(\begin{array}{cccc} -10 & 10 & 0 & 0\\ -10 & -1 & 0 & 0\\ 0 & 0 & -\frac{8}{3} & 0\\ 0 & 0 & 0 & -1 \end{array}\right)$. Therefore, *λ*_±_ = −5.5 ± 8.9303*j*, *λ*_3_ = −8/3, *λ*_4_ = −1, and -max⁡[Reλ(L+FΩ)]=1>[Γ(q)]1/q=0.9722, respectively. Simulation results are shown in Figure 3. Here, *b*(0) = 10.

**Case 3 (parameter *c* is unknown in response system).**

Now, assume that the system parameter  *c*  in response system is the unknown parameter. The true value of the “unknown” parameter  *c*  is selected as  *c*_0_.

The fractional-order Lorenz system (12) can be rewritten as

(29) $$\begin{array}{c} \left(\begin{array}{c} D^{q}x_{1}\\ D^{q}x_{2}\\ D^{q}x_{3} \end{array}\right)=\left(\begin{array}{cccc} -10 & 10 & 0 & 0\\ 28 & -1 & 0 & 0\\ 0 & 0 & 0 & 0 \end{array}\right)\left(\begin{array}{c} x_{1}\\ x_{2}\\ x_{3}\\ c_{0} \end{array}\right)+\left(\begin{array}{c} 0\\ -x_{1}x_{3}\\ x_{1}x_{2}-c_{0}x_{3} \end{array}\right). \end{array}$$

So

(30) $$\begin{array}{c} L=\left(\begin{array}{cccc} -10 & 10 & 0 & 0\\ 28 & -1 & 0 & 0\\ 0 & 0 & 0 & 0 \end{array}\right). \end{array}$$

According to Section 2, the response system is given as

(31) (Dqy1Dqy2Dqy3)=(−10100028−1000000)(y1y2y3c)+(0−y1y3y1y2−cy3)+(F−nlp(x))(e1e2e3ec)

and the parameter update law is

(32) $$\begin{array}{c} D^{q}c=\Omega {\left(\begin{array}{cccc} e_{1} & e_{2} & e_{3} & e_{c} \end{array}\right)}^{T}. \end{array}$$

It is easy to obtain the following:

(33) (0−y1y3y1y2−cy3)−(0−x1x3x1x2−c0x3)  =(0000−x30−x10x2x1−c0−x3)(e1e2e3ec)   +(0−e1e3e1e2−ece3).

So

(34) nlp(x)=(0000−x30−x10x2x1−c0−x3),nnp(e,x)=(0−e1e3e1e2−ece3).

Now, it is easy to verify the following:

(35) ||(nnp(e,x)0)||||e|| =(e1e2−ece3)2+(e1e3)2e12+e22+e32+ec2 ≤(|e1||e2|+|ec||e3|)2+(e1e3)2e12+e22+e32+ec2 =(2|e1||e2||e3||ec|e12+e22+e32+ec2+(ece3)2e12+e22+e32+ec2   +(e1e2)2+(e1e3)2e12+e22+e32+ec2)1/2 ≤2|e1||e2||e3||ec|e12+e22+e32+ec2+(ece3)2ec2+(e1e2)2+(e1e3)2e12 =2|e1||e2||e3||ec|e12+e22+e32+ec2+2e32+e22 ≤2|e1||e2||e3||ec|2|e1||e2|+2|e3||ec|+2e32+e22 =(1|e3||ec|+1|e1||e2|)−1+2e32+e22,lim⁡e→0||(nnp(e,x)0)||||e||≤lim⁡e→0(1|e3||ec|+1|e1||e2|)−1+2e32+e22=0,(nnp(e,x)0)|e=0=0.

Therefore, the first condition in the above-mentioned theorem holds.

Now, choose suitable real constant matrix *Ω* ∈ *R* ^1×*n*^ and *F* ∈ *R* ^*n*×(*n*+1)^ such that

(36) Re[λ(L+FΩ)]<0,−max⁡[Reλ(L+FΩ)]>[Γ(q)]1/q.

So, the second condition in the above-mentioned theorem holds. According to the theoremin Section 2, the adaptive synchronization between drive system (29) and response system (31) with parameter update law (32) can be achieved.

**Figure 4:**
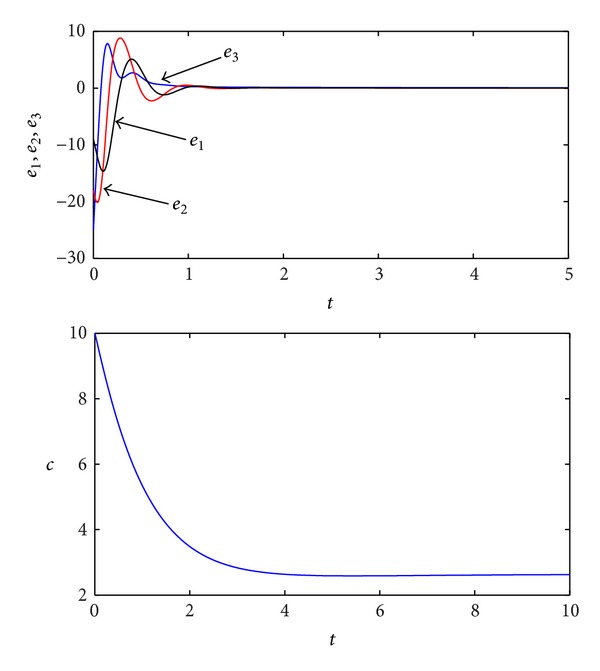
Simulation results of the adaptive synchronization for the fractional-order Lorenz chaotic system.

For example, let $F=\left(\begin{array}{cccc} 0 & 0 & 0 & 0\\ -38 & 0 & 0 & 0\\ 0 & 0 & -1 & 0 \end{array}\right)$ and $\Omega =\left(\begin{array}{cccc} 0 & 0 & 0 & -1 \end{array}\right)$. So $\left(\begin{array}{c} L+F\\ \Omega \end{array}\right)=\left(\begin{array}{cccc} -10 & 10 & 0 & 0\\ -10 & -1 & 0 & 0\\ 0 & 0 & -1 & 0\\ 0 & 0 & 0 & -1 \end{array}\right)$. Therefore, *λ*_*i*_ = −1  (*i* = 1,2), *λ*_±_ = −5.5 ± 8.9303*j*, and -max⁡[Reλ(L+FΩ)]=1>[Γ(q)]1/q=0.9722, respectively. Simulation results are shown in Figure 4. Here, *c*(0) = 10.

## 4. Conclusions

One adaptive synchronization scheme for a class of fractional-order chaotic system with fractional-order 1 < *q* < 2 is suggested in this paper. This synchronization approach is based on a new stability result of equilibrium point in nonlinear fractional-order systems for fractional-order lying in 1 < *q* < 2. In order to verify the effectiveness of the adaptive synchronization approach, the adaptive synchronization for the fractional-order Lorenz chaotic system with fractional-order 1 < *q* < 2 is considered. Numerical simulations show the validity and feasibility of the proposed scheme. The current results in this paper can be extended to several unknown parameters of the fractional-order chaotic systems. Moreover, this synchronization approach can be applied to other fractional-order chaotic systems.
